# Crops on the Rocks: Production, Processing, and Storage at the Early Medieval Site of Senhora Do Barrocal (Municipality of Sátão, Central Portugal)

**DOI:** 10.3390/plants11040471

**Published:** 2022-02-09

**Authors:** Luís Seabra, Catarina Tente, Filipe Costa Vaz, Cláudia Oliveira, Lara González Carretero, João Pedro Tereso

**Affiliations:** 1 CIBIO, Research Center in Biodiversity and Genetic Resources, InBIO Associate Laboratory, Campus de Vairão, University of Porto, 4485-661 Vairão, Portugal; lc_pacos@hotmail.com (L.S.); filipe.mcvaz@gmail.com (F.C.V.); 2 BIOPOLIS Program in Genomics, Biodiversity and Land Planning, CIBIO, Campus de Vairão, 4485-661 Vairão, Portugal; 3 Institute for Medieval Studies, NOVA University of Lisbon, Av. Berna 26C, 1069-061 Lisboa, Portugal; catarina.tente@fcsh.unl.pt; 4 Interdisciplinary Laboratory for Continental Environments, Campus Bridoux, University of Lorraine, Rue du Général Delestraint, 57070 Metz, France; claudia.oliveira1991@gmail.com; 5 The British Museum, Department of Scientific Research, Museum of London Archaeology (MOLA), London WC1B 3DG, UK; lgonzalezcarretero@mola.org.uk; 6 Centre for Archaeology, UNIARQ, School of Arts and Humanities, University of Lisbon, Alameda da Universidade, 1600-214 Lisboa, Portugal; 7 MHNC-UP, Natural History and Science Museum of the University of Porto, Campo dos Mártires da Pátria, 81, 4050-368 Porto, Portugal

**Keywords:** fruits/seeds, charcoal analysis, food remains, Middle Ages, western Iberia

## Abstract

Small rural places are largely absent from early medieval written sources, but they were profuse and relevant in regional settlements and economies. Only through archaeological and archaeobotanical investigation is it possible to unveil their structure and productive strategies; however, this kind of investigation is still uncommon in Iberia. Here, the assemblage of fruits/seeds, wood charcoal, and food remains from Senhora do Barrocal (SB) (Sátão, Portugal) will be presented and discussed in order to understand the crop production, processing, and storage. The site was destroyed by a fire somewhere between the 10th and the 11th centuries AD, which allowed the preservation of abundant plant remains in a storage area. Charcoal analyses suggest that the building was made with oak and chestnut timber. The massive fruits/seeds assemblage was dominated by cereals, mostly oat and rye, but also barley, millet, and naked wheat, some fully dehusked, others still hulled. Furthermore, evidence of food products has also been found, suggesting that the area was used for the storage of multiple foods and crops at different processing stages. SB is a good example of how communities adopted a diverse set of crops and multifaceted storage strategies to prevent food shortages and to endure in a harsh environment.

## 1. Introduction

The early Middle Ages was a complex period of instability in Iberia, particularly due to the Muslim conquest and the incorporation of most of the peninsula’s territory in the Umayyad Caliphate in the beginning of the 8th century AD. From the 8th to the 11th centuries, the area that is now central Portugal became a frontier between the Christians and the Muslims and subject to military campaigns and power shifts (e.g., [1,2]). The definitive Christian conquest of the region only took place in the mid-11th century, following the collapse of the Cordoba Caliphate amidst internal turmoil [1,2,3,4].

This warring period and the inherent political instability permeated throughout this area and its communities. However, such conditions allowed them to shape and define their own territories, configuring a fragmented and unstructured landscape, as they could freely explore local resources and establish their own internal and external relations [5,6]. During these turbulent times, rural communities kept the daily and seasonal activities which their survival depended on, where the exploitation of natural resources assumed a great relevance.

The considerations regarding early medieval agriculture in western Iberia are still scarce and usually rely on written sources from later periods [7,8]. Moreover, those historical documents are mostly property transactional contracts, donations, wills, and *foral* charters, which reflect the direct or indirect interests of the elite and are not representative of the daily life of much of the rural communities of that time. References regarding the use of other resources are virtually absent from the written sources. Wood, for instance, was a crucial raw material for medieval communities, being the preferred construction material for housing and other facilities, as well as the main fuel for everyday activities. Thus, its gathering strategies and multiple uses had important economic and social roles for medieval societies.

In central Portugal, the earliest written sources that mention agricultural activities/products are from the 12th–13th centuries AD; earlier references are scarce. At the municipality of Sátão (Viseu), two *foral* charters, dated from 1126 (Ferreira de Aves) and 1149 (Sátão), mention some goods that were used for tax payments to the central authority, such as wheat, millet, and barley, but also wine, flax, honey, and faba beans [7]. Despite their value in characterizing the relationship between the rural communities and the dominant power, these charters do not reveal the full extent of the agricultural practices and food consumption of these communities as they only focus on taxable goods.

Archaeological and archaeobotanical data help decisively to bridge the gap between the written documentation and the everyday activities of these communities. These approaches become even more relevant in regions and historical periods where written accounts are rare, as is the case for this region. In fact, the archaeobotanical analyses are fundamental to understanding the agricultural practices and storage strategies as well as the use of wood for multiple purposes. This has been tried and tested for other areas of the Iberian Peninsula as well as of Western Europe (e.g., [9,10,11,12,13]). However, archaeobotanical investigation in medieval Iberia is recent and spatially heterogeneous, which contrasts with other chronological periods [14,15].

**Figure 1:**
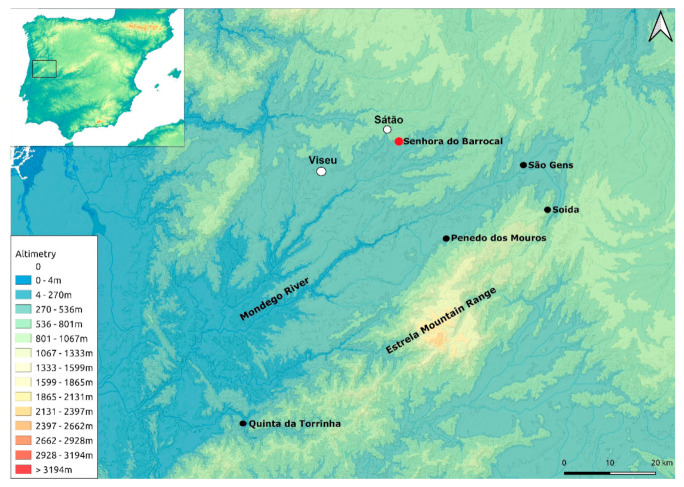
Location of Senhora do Barrocal (SB) and other sites in central Portugal with fruits/seeds.

In western Iberia, some studies have been carried out on isolated sites or in relation with regional archaeological projects (e.g., [15,16,17,18]). In central Portugal, namely the Mondego basin, plant remains were recorded in few medieval rural sites (Figure 1). Archaeobotanical studies have been conducted at Soida (Celorico da Beira) [19], Penedo dos Mouros (Gouveia) [20,21,22], São Gens (Celorico da Beira) [14,23], and Quinta da Torrinha (Goís) [24], but most of them provided few remains. Thus, the development of more analyses is fundamental.

Recent archaeological campaigns carried out at Senhora do Barrocal (SB) (Figure 1) revealed one of the largest assemblages of charred plant remains from the Middle Ages in Portugal, preserved by a fire that destroyed a domestic structure. Previous and preliminary archaeobotanical analysis of a part of the assemblage has already confirmed the potential of the site [25,26]. This paper will present the full results of the fruits/seeds and charcoal analyses, as well as the identification of the food remains, and integrate them into the site archaeological record. It will focus on its space organization and construction, the storage practices, and the role of crops and wild plants in this community’s economy. The results will also be interpreted together with other archaeological and historical data to evaluate the agricultural choices and the use of plant resources at SB, in the context of the political, cultural, and landscape history of western Iberia.

## 2. The Medieval Site

**Figure 2:**
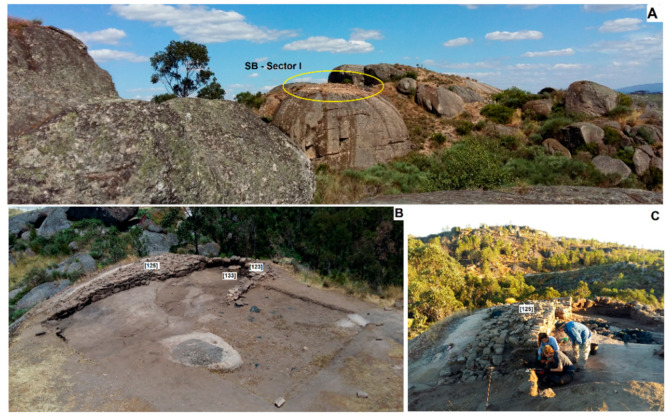
Sector 1 implantation (**A**); Sector 1 overview (**B**); wall 112 dismantling (**C**).

The archaeological site of SB (40°44′17.80″ N/7°38′32.64″ W) is located in the Portuguese municipality of Sátão in the district of Viseu (central Portugal). It was implanted on the top of a prominent granitic tor (Figure 1 and Figure 2A), at 598 m a.s.l., near the fertile valley of the Côja stream (located at approximately 1.5 km), a tributary of the Dão river, both being part of the Mondego river basin. The site’s location provides visual control over the valley while remaining relatively unnoticed on this hilly granite landscape.

**Figure 3:**
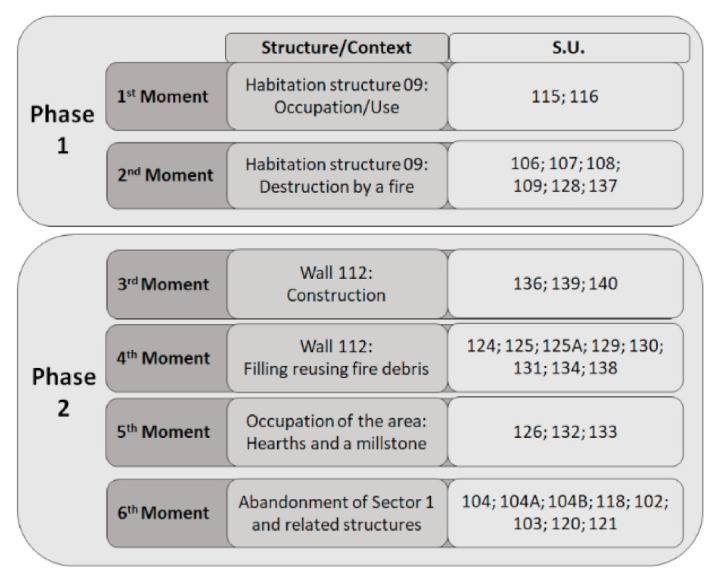
Relation between the archaeological contexts, structures, and site phasing.

Here, three archaeological campaigns were developed between 2014 and 2016, in two sectors. In Sector 1, two occupation phases were identified, separated by a large layer of ash and charcoal resulting from a fire which destroyed most of the standing structures. In Sector 2, a single level of occupation was observed, showing few archaeological and archaeobotanical remains. For that reason, this sector is not addressed in this study. Each occupation phase of Sector 1 was subdivided into different moments (Table S1, Figure 3), which are described as follows:

Phase 1—The first moment of occupation is characterized by a level of circulation (stratigraphic units (s.u.) 115 and 116) inside the domestic structure 09, built with perishable materials. Here, it was only possible to recover some domestic goods. A fire (second moment) destroyed this structure, marking the end of the first phase at SB. Associated with this destructive event are several s.u. (Table S1, Figure 3), which revealed debris from the building and its fillings, including several artefacts (e.g., metals, pottery, and beads). Nonetheless, the fire allowed the preservation of a massive assemblage of archaeobotanical remains.

Phase 2—This corresponds to the occupation that followed the fire, during which the site suffered some changes. At first, and corresponding to the site’s third moment, the stone wall 112 (Figure 2B) was built/rebuilt over the ashy layer from the previous phase’s fire. This stratigraphic relation was testified after dismantling a part of the wall (Figure 2C). Larger stones were used to define the wall facings (inner and outer), whereas its core was filled (fourth moment) with varied materials, such as small stones, gravel, or even debris from the fire. Several layers are related to the latest moment, including broken ceramic vessels (s.u. 124) and sediments with archaeobotanical material (s.u. 125, 125A, 127, 130, 131, 134). Wall 112 showed two construction stages, likely separated by a short period of time and corresponding to the use of different techniques [26]. Remains of the fire are found beneath both.

Other stone structures were scarce. Leaning against wall 112, the stone structure 123 (Figure 2B) (fifth moment) was an exception, but its functionality was not fully understood. This structure was perhaps part of a house, which could have been delimited by the wall. Associated with the site’s fifth moment were also two hearths (s.u. 126 and 132), as well as a broken granite millstone (s.u. 133) (Figure 2B), which was taken from a previous stage and reused during this moment. The sixth and last moment corresponds to the abandonment of the structures from Phase 2 and consequently of the site (Table S1, Figure 3).

Despite the effort applied to constructing the wall, the second occupation of SB probably did not last long. In fact, both of the occupation phases can be dated between the 10th and 11th century AD [2,26]. The establishment of this chronology included archaeological artefacts, radiocarbon dates, and an inscription. Moreover, the SB occupation could be related to other archaeological sites in the region, which revealed similar chronologies [2]. Two of the three radiocarbon dates (Table 1) are related to the fire that led to the collapse of the Phase 1 building, namely the faba bean (*Vicia faba*) from destruction layer s.u. 107 and the grain of rye (*Secale cereale*) from s.u. 125, a sediment with abundant plant remains which was remobilized and used to fill wall 112 afterwards. The third radiocarbon date was obtained from a tree heath charcoal (*Erica arborea*) recovered in a clay layer (s.u. 132) from Hearth 2. It is stratigraphically associated with the second phase of occupation (fifth moment), after the fire.

The radiocarbon analysis results, however, do not point to clear differences between the two phases, especially those from s.u. 107, and 132, which are probabilistically similar and non-discernible. In fact, these dates strengthen the idea that after the fire (Phase 1), the occupation at SB did not last long. An inscription from 971 AD should also be considered. It was found inside a modern church, which was probably built in the same place of a former building and just a few meters away from the site. Its location and date suggest that both SB and the ancient church were related [2,26,29]. Despite it not being possible to exclude other chronologies, considering the long time span revealed by the *Secale cereale* date (772–974 cal. AD), the church was likely built before the fire, suggesting that the destruction occurred between the last quarter of the 10th century and the first two decades of the 11th century AD [2,25,26].

Moreover, the data from SB, although with their own peculiarities, fit well with the dynamics recorded in other rural sites in the Mondego region, namely Soida, Penedo dos Mouros, and São Gens. Besides some structural similarities, these were all destroyed by fires around the 10th century AD, as suggested by the radiocarbon dates [2,30]. In contrast with SB, these sites were not reoccupied. There is, thus, a regional trend, likely connected with the new political order that was emerging at that time (see above).

The archaeological data from SB show that its inhabitants had diverse contacts, at a local sphere and beyond. The presence of exogenous pottery, uncommon in the region, demonstrates that SB would have been regionally relevant [2,26]. Still, it is quite challenging to evaluate the level of engagement of this community in the regional and wider conflicts of the time and how these constrained them. Instead of a complete integration of Christian or Muslim rules, the archaeological data from SB, as well as the panorama identified in the region by the end of the 10th century, suggest some level of local engagement by prominent elites, who took advantage of the conflict and tried to control the region [2].

## 3. Materials and Methods

### 3.1. Sampling Strategy

Sampling and recovery strategies varied throughout the three archaeological campaigns at SB (2014–2016) (Table 2 and detailed information in Table S1). In 2014, plant remains were handpicked and recovered through dry sieving. A single sediment sample was floated with a 0.5 mm mesh. Although this resulted in a recovery bias, neglecting the small plant remains, considerable concentrations of charred remains were recovered. The fieldwork was restricted to a small area and the deposits related to the major fire were identified but practically not excavated.

In the following campaigns, the sampling, recovery, and processing strategies were gradually adjusted to the current practices. Sediment samples were processed through bucket flotation (2015) or with a Siraf-type flotation machine (2016), using 0.5 mm meshes, in combination with handpicking. Volume was not always recorded but, in 2016, each sediment sample associated with the fire level (second moment), the wall construction (third to fourth moments), and the abandonment (sixth moment) corresponded to c. 10 litres (a full bucket) (see Table S1). Using a flotation machine allowed the processing of more sediment and, consequently, the recovery of a higher amount of archaeobotanical remains.

Due to this large number and the volume of samples processed in the 2016 campaign, it was not possible to study the entire assemblage. Five hundred and forty-six samples were recovered from 30 s.u., 439 (80%) of which were analysed, including samples from all the stratigraphic units and 92 squares (1 m^2^ each) in Sector 1 (Table S1). Selection was carried out using the Microsoft Excel (Microsoft Corporation, Redmond, WA, USA) random number generator, assuring that at least one sample per each of the possible s.u./square combinations was studied. As the decision for adopting this procedure occurred only when some sediment samples from 2016 were already analysed, there are minor discrepancies in the amount of samples studied per s.u./square combination.

Eight contexts from Phase 1 were sampled. The first moment is represented by 15 sets of handpicked remains from s.u. 115 and 116. From the second moment of occupation, between the handpicked remains and sediment samples, 263 samples were recovered in six s.u. Most samples (207) were recovered in s.u. 107, where large concentrations of plant remains were visible, resulting from the fire that led to the destruction of the building. The samples from this s.u. were recovered in 34 different squares, this being the most extensive layer in the whole site.

The sampled contexts from the third and fourth moments belong to Phase 2. These are associated with the building (preparation/construction) and filling of wall 112, but most of the remains associated with this phase are the result of the remobilization of sediments from the fire of the previous phase. Related to these two events are 132 samples, mostly from a filling layer (s.u. 125) of wall 112 (Table S1).

The fifth moment is represented by two hearths and a granite millstone where four sediment samples and an equal number of handpicked remains were recovered.

From the final abandonment levels of Sector 1 and the related structures (sixth moment), eight stratigraphic units were sampled. However, most of the samples (120 out of 128) correspond to sets of handpicked remains. All the samples from this moment were analysed Table 2 and Table S1).

### 3.2. Laboratory Analysis

#### 3.2.1. Fruits/Seeds

The flotation fractions were sorted using a stereoscopic microscope, during which the fruits/seeds were identified by comparison with morphological atlases and other specialized bibliographies (e.g., [31,32,33,34,35,36,37,38]) and with modern material from the reference collections of the Porto Herbarium (PO) at the Natural History and Science Museum of the University of Porto (MHNC-UP) and from the Research Centre in Biodiversity and Genetic Resources (CIBIO).

To facilitate the sorting, the flotation fractions were separated using a column of sieves with 2 mm, 1 mm, and 0.5 mm meshes. Due to their large volume, the contents of some of the meshes from 50 samples were subsampled with a riffle box (Table S2). In such cases, the results presented here are extrapolations that take into consideration the percentage of the flotation fraction that was analysed. Extrapolation was only applied to the units.

**Figure 4:**
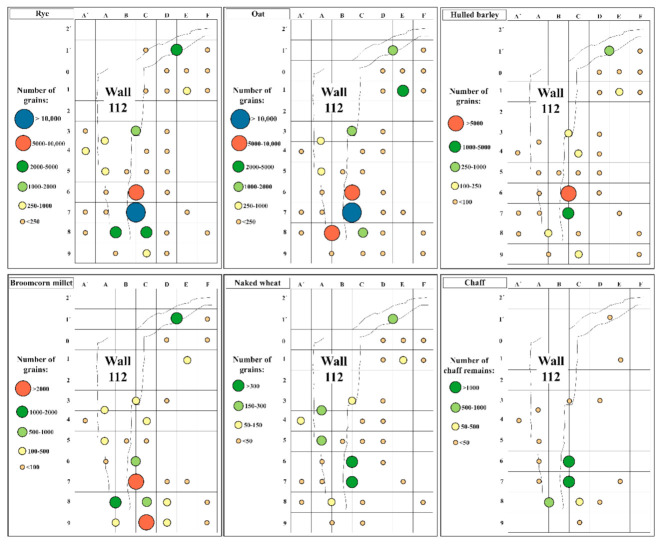
Individual results of the main cereal grains and chaff per square in Sector 1 (total results); only units. Foxtail millet grains were scarce, and for that reason are not graphically represented. Results from the first moment as well from row G onwards revealed few remains and for that reason are not included.

In this paper, whole fruits/seeds and fragments with scutella or hila were counted as units. In the case of the longitudinal fragments (cereals) and cotyledons (pulses), two remains were counted as one unit. The results including units only are displayed in Table S3, while the full fruits/seeds results (both units and fragments) per sample and square/s.u. are presented in Tables S4 and S5, respectively. Regarding chaff, although inherently fragmentary, the spikelet bases, floret bases, lemma bases, and rachis segments as well as rachis nodes were considered as units. When grains were found attached to the lemma bases (hulled grain) or with husks, but without the base, they were discriminated in the full results (see Tables S4 and S5), whereas in Tables S3 and S6, as in Figure 4 and Figure 6, the chaff and grains were always treated separately, even when they were found attached. A similar procedure was applied to the aggregated cereals.

Taxonomic nomenclature follows the International Plant Name Index [39], but due to space limitations, shorter designations, excluding author names, will be used in Table 3 and throughout the text. For a complete nomenclature see Supplementary Tables S3–S6. The designation *Avena sativa/strigosa* was applied whenever the floret bases were present, according to Jacomet [35], Van Zeist [40], Pasternak [41], and Ruas and Pradat [42]. The distinction between both oats is often difficult, and in this study was not achieved unequivocally. Despite this, the features observed may allow some approximation (see crop diversity). The grains without floret bases, whether naked or with fragmented husk remains, were identified at the genus level (*Avena* sp.). For naked wheat, the designation *Triticum aestivum/durum* was adopted, following Buxó [34], which includes *Triticum aestivum*, *Triticum durum*, and *Triticum turgidum* grains.

#### 3.2.2. Charcoal Analysis

In SB, the charcoal analysed was recovered in handpicked samples and in the 2 mm sieve of the floated samples. The charcoal fragments were sectioned manually in order to obtain the cross, tangential, and radial diagnostic sections. Their anatomical characteristics were examined using a reflected light microscope and compared with several anatomical atlases (e.g., [43,44]), with PO and from the CIBIO reference collection. As indicated for the fruits/seeds analysis, the taxonomic nomenclature followed the International Plant Name Index [39]. Complete designations are included in Supplementary Tables S7–S9, whereas, in Table 4, the designations do not include the author names. In the case of the genus *Erica*, the criteria described by Queiroz and van der Burgh [45] and adapted by Tereso [46] was followed instead.

A minimum of 100 charcoal fragments analysed per sample was initially set for this study, taking into account the type of context and sampling strategy devised, with multiple samples recovered from the same s.u. However, when a new taxon was identified in the last 50 analysed charcoals in the sample, an additional 50 fragments underwent analysis. This was performed successively until no new taxa were identified, sometimes resulting in a number of fragments identified per sample higher than the referred 100 charcoal fragments.

Dendrological characteristics and anatomical alterations were recorded for each charcoal fragment in order to obtain taphonomic and palaeoethnobotanical data, including, among others, tree-ring curvature [47], the presence of vitrification [48], radial cracks [49], and biodegradation [50].

While performing the spatial analysis only the results obtained from the floated samples with the minimum threshold of 100 charcoal fragments identified were used, in order to assure comparability of the data.

#### 3.2.3. Analysis of Food Remains

Several fragments of amorphous charred matter were identified during the sorting of the archaeobotanical material as potential fragments of cooked food, specifically cereal products, such as bread or porridge-like foodstuffs. Studies concerning these kinds of remains have been thoroughly conducted in the last ten years, following different methods and over the remains of several chronologies (e.g., [51,52]).

In order to confirm the initial identification, six charred fragments were analysed using a range of microscopic techniques. As a first step, the selected fragments were examined under a Leica EZ4 binocular microscope at magnifications of 8×/40×. Photographs of the fragments surfaces were captured using a Leica S6D microscope and a Leica EZ3 camera (Leica Microsystems, Solms, Germany) in order to keep a record of the fragments’ macromorphology and external physical characteristics. Consequently, the amorphous charred fragments were analysed under a Scanning Electron Microscope (SEM) with the purpose of observing their internal microstructures as well as identifying any potential components or particles embedded. Samples were cleaned from the soil and concretion and prepared following standard procedures for SEM observation. Once prepared and mounted in aluminium stubs, the samples were examined using a Hitachi S–3700N SEM (Hitachi Ltd., Chiyoda, Tokyo, Japan) at the Scientific Research Department at the British Museum. The SEM study of the selected fragments followed the methods outlined in González Carretero et al. [53] and González Carretero [54] for the high-resolution analysis of the fragments’ microstructures and the detailed examination of the porosity and visible particles.

## 4. Results

### 4.1. Fruits/Seeds

**Figure 5:**
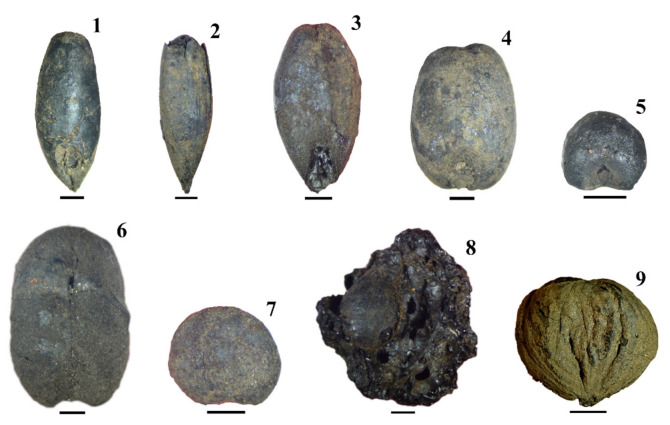
Some of the fruits/seeds found at SB: 1—rye grain; 2— Hulled grain of oat; 3—barley grain with husk remains still attached; 4—naked wheat grain; 5—broomcorn millet grain; 6—faba bean seed; 7—lentil seed; 8—berry and pip of grape; 9—chestnut fruit. With the exception of chestnut (5mm scale), all remains show a 1 mm scale.

The results show an overwhelming assemblage of fruits/seeds, mostly cereal grains (90.6%) (Figure 4 and Figure 5, Table 3 and Tables S3–S5). Among these, grains of oat (*Avena* sp. and *Avena sativa/strigosa*) and rye (*Secale cereale*) were predominant. They were identified in similar quantities, but the volume of rye is higher due to the larger size of the grains. Both represent 64% of all the fruits/seeds collected. Grains of hulled barley (*Hordeum vulgare*), broomcorn millet (*Panicum miliaceum*), and naked wheat (*Triticum aestivum/durum*) were found in smaller amounts. A few grains of foxtail millet (*Setaria italica*) were also identified.

Chaff comprises only 2.6% of all the plant remains, but rye rachis segments, oat floret bases, and barley lemma bases were found in several layers, particularly where the grains were more common. Other remains, such as rachises from naked wheat and barley, as well as millet grains with lemma/palea, were also collected, but in fewer amounts and in less layers (Table 3 and Table S3–S5).

Besides cereals, cultivated pulses were also found, namely seeds from pea (*Pisum sativum*), faba bean (*Vicia faba*), grass/red pea (*Lathyrus cicera/sativus*), and lentils (*Lens culinaris*). Pea and faba bean were slightly more abundant than grass/red pea and lentils (Figure 5). Despite its poor preservation, *Vicia/Lathyrus* and *Vicia/Lathyrus/Pisum* seeds may also correspond to cultivated legumes. It is relevant that some diversity of pulses was identified, although both cultivated and wild legumes amounted to just 1.6% of the fruits/seeds assemblage.

Fruits were almost absent (0.1% of the assemblage). Nevertheless, some diversity was recorded. Pips, pedicels, and two whole berries of *Vitis vinifera* (Figure 5) were found. Chestnut fruits (*Castanea sativa*) (Figure 5), sweet/sour cherry endocarps (*Prunus avium/cerasus*), bract fragments of cluster/stone pine (*Pinus pinea/pinaster*), a possible pear seed (*Pyrus* sp.), and fragments of *Quercus* sp. acorns were collected but always in small amounts. The interpretation of some of those remains as cultivated or wild should be carefully addressed (see below).

The results provided a single oil/fibre taxon. Six agglomerates of flax (*Linum* sp.) seeds were identified in s.u. 107. Their state of preservation did not allow a more precise identification; still, they likely correspond to cultivated flax, namely to *Linum usitatissimum*.

Wild Poaceae, including *Festuca/Lolium*, among others, were frequent, comprising 3.6%. Other wild plants, such as cornockle (*Agrostemma githago*)*,* scarlet pimpernel (*Anagallis arvensis*)*,* cleavers (*Galium aparine*), ribwort plantain (*Plantago lanceolata*)*,* wild radish (*Raphanus raphanistrum*), field madder (*Sherardia arvensis*), common catchfly (*Silene gallica*), or corn spurrey (*Spergula arvensis*), accounted for only 1.5% of the fruits/seeds assemblage. Those remains are common weeds, although they can also be found in other ruderal contexts [32,55].

**Figure 6:**
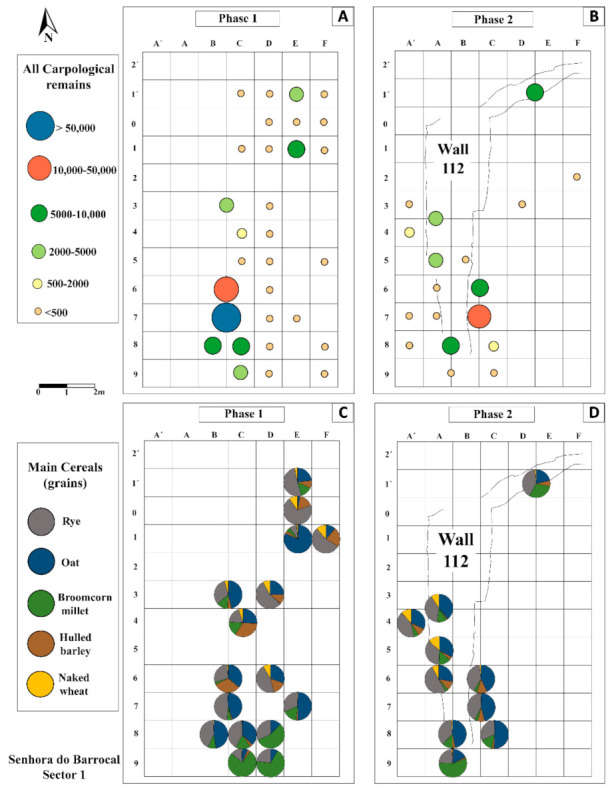
Square analysis per phase. All fruits/seeds (**A**,**B**); main cereal grains (**C**,**D**); only units. Results from the first moment as well from row G onwards revealed few remains and for that reason are not included.

The fruits/seeds assemblage varied between the two phases and, above all, within the six moments of occupation (Table 3 and Tables S3–S5 and Figure 6). The first moment in Phase 1 (s.u. 115 and 116) revealed few fruits/seeds (n = 184), but it is necessary to take into consideration that all the samples from these layers were handpicked. This small assemblage does not allow a proper analysis and integration in the site results.

The second moment, corresponding to the great fire that destroyed building 09, is particularly relevant to this study as most of the remains found came from this event. Six layers were studied, but s.u. 107 comprised 99.2% of the remains from this phase and 70.2% of the remains collected in the whole site. A closer analysis of s.u. 107 shows that 85.1% of its fruits/seeds were recovered within 7 m^2^, in squares B6-C6-B7-C7-B8-C8-C9 (Figure 6A, Tables S4 and S5). It is noteworthy that around half of those remains were collected in just two squares—B7 and C7.

The concentration of fruits/seeds in such a confined space is particularly relevant, but s.u. 107 also concentrated most of the sampling effort. In 2016, the use of the flotation machine allowed the recovery of more sediment samples, mainly in this layer and in square C7 (27 sediment samples). Laboratory work also gave particular emphasis to this s.u./square combination. However, despite the discrepancies in the number of samples analysed in each s.u. and square, the data regarding the units per Litre (units/L) suggest there really was a higher concentration of fruits/seeds in s.u. 107, square C7. In fact, considering that each sample recovered in 2016 comprised around 10 L of sediment (see Materials and Methods and Table S1), the results show that the five samples (≈50 L) from square C7 comprise around 919 units/L. However, we must emphasize that just one sample (185) has revealed 2400 units/L, which is more than double that of any other sample. The same s.u. 107 provided smaller concentration of remains in other squares, such as B8 (661 units/L) and C9 (430 units/L). Outside the area where most of the remains were retrieved, a few other squares also revealed relevant concentrations, such as E1 (513 units/L) or E1’ (331 units/L) (see details in Table S6).

More patterns in s.u. 107 were identified (Tables S4 and S5 and Figure 6C): grains of oat (19,714 out of 36,609) and rye (21,032 out of 34,204) were more abundant in squares B7/C7; broomcorn millet was mostly concentrated in squares C9 (2673 out of 8345) and B7 (1374 out of 8345); but grains could be found all over the area. There were great concentrations of grains from the hulled barley in B6/C6 (5511 out of 7248), where we also found almost all the barley chaff found in the settlement so far (733 out of 913); flax agglomerates were found in squares F1’ and F0, where other fruits/seeds were scarce. However, some caution is required as the samples from these two squares correspond only to the handpicked remains (Tables S1, S4 and S5). In the other fire levels, the fruits/seeds were scarce, which is not surprising as most of those samples correspond to the handpicked materials.

After the fire, Sector 1 suffered some changes. Compared with the previous phase, the amount of fruits/seeds was much smaller (n = 45,266) in Phase 2 (Tables S3–S5 and Figure 6B). However, there was a significant amount of fruits/seeds in some layers, associated with the fourth moment, namely in s.u. 125 (n = 14,587) and 130 (n = 11,677), which are the filling deposits of wall 112. The same happened with s.u. 104 (n = 6661), from the sixth moment, which is also related to wall 112, but in this case to its abandonment (Tables S3–S5). The structures associated with the fifth moment revealed few remains, and no differences were observed in the fruits/seeds assemblage.

As in the earlier phase, Phase 2 was also dominated by grains, mostly rye and oat. Together, these represent more than half (n = 27,671) of the remains found. Considerable concentrations of fruits/seeds came from some of the squares already mentioned (B6-C6-B7-C7), or next to them (e.g., A8/B8, D1’-E1’).

The estimated volume also attests to the observed trend, being the amount of units/L smaller than in Phase 1 (Table S6). Despite this, exceptions were observed in some of the abovementioned squares and layers. One sediment sample (358) in B7 from s.u. 130 showed a really high concentration of remains (869 units/L) and again from square B7, but in s.u. 125, another sample (348) revealed a considerable concentration (338 units/L) of fruits/seeds (Table S6). In D1’-E1’ from s.u. 104, two sediment samples also showed a significant concentration (333 units/L), taking into account an estimated volume of 20 litres. These samples strengthen the importance of some squares with regard to the space organization of Sector 1. 

Besides the smaller amount of fruits/seeds recovered in the Phase 2 contexts, substantial differences between this and the previous phase were not perceived (Tables S3–S5 and Figure 6), which may reflect the use of sediment with charred plant remains from the fire that occurred at the end of Phase 1 in the filling of the walls built in Phase 2.

### 4.2. Charcoal Analysis

A total of 18,601 charcoal fragments were identified at SB. Phase 1 provided a diverse set of taxa (Table 4). *Castanea sativa* was the most frequent with 34.2% of the total, followed by *Quercus* sp. deciduous (16.1%) and *Castanea/Quercus* (11.4%). All the Fagaceae combined sum up to 64% of the charcoal analysed. A second group was also relevant, which included *Erica australis/arborea* (5.9%), *Arbutus unedo* (6%), *Erica* sp. (4.2%), and *Prunus* sp. (3.7%). The remaining 16 taxa comprised just 7.3%. Undetermined and dicotyledon fragments accounted for almost 11%. Despite 85.9% of all the samples from Phase 1 being handpicked, the results from the floated samples confirm the diversity and distribution of the taxa observed.

The results from Phase 2 displayed similar results to those of Phase 1, which is not surprising considering that their origin is most likely the same. Among the 8765 charcoal fragments identified, *Castanea sativa* was still, by far, the most frequent, with more than 1/3 of the total (35.8%), followed by *Quercus* sp. deciduous (22.7%). As before, a second group of taxa also displayed a reasonable percentage, such as *Arbutus unedo* and *Erica australis*/*arborea* (4.5% and 4.9, respectively), *Prunus* sp. (4%), *Erica* sp. (3%), and Fabaceae (2.2%). All remaining taxa only accounted for 5.2%, with the exception of *Castanea/Quercus* (6.8%) and dicotyledons which had the same percentage as in Phase 1 (c. 10.7%).

Regarding the tree-ring curvature, the shrubs displayed higher percentages of moderate and strong ring curvatures: *Arbutus unedo* (57%), *Cistus* sp. (32%), *Erica* spp. (from 56% to 67% according to taxon), and Fabaceae (84%). On other hand, the arboreal taxa displayed weak tree ring curvatures: *Alnus* sp. (87%) and *Fraxinus* sp. (76%), but especially *Castanea sativa* (80%) and *Quercus* spp. (from 68% to 70%).

Vitrification was mostly concentrated in dicotyledons (68% of the fragments from this taxon) and undetermined charcoal (89%), which in most cases explains such undetailed identification. The remaining taxa, with the exception of *Quercus* sp. and *Erica* spp., displayed less than 10% of the vitrified fragments. In contrast, radial cracks were recorded in dicotyledons (35% of the fragments from this taxon), *Erica* spp. (from 14% to 31%), Fabaceae (30%), *Quercus* spp. (from 13% to 27%), and *Cistus* sp. (19%).

As previously stated, the spatial analysis of these results only took into account the floated samples from Phase 1, with a minimum of 100 charcoal fragments identified. This exercise revealed an even distribution of *Castanea sativa* and *Quercus* sp., along with most other taxa, throughout the excavated area. The exception to this trend was a small concentration of *Prunus* spp. charcoal identified in squares D9 and F9 in the western part of the area. The full anthracological results are available in Supplementary Tables S7–S9.

### 4.3. Food Remains

The six amorphous charred fragments ranged from 1 cm to 4.2 cm in size and their degree of charring varied from partially charred to fully charred. Macroscopically, these remains were preliminarily identified as potential fragments of cooked food, such as bread-like or porridge-like products, due to their amorphous, porous, and homogeneous appearance as well as their starchy internal structure (Table S10).

**Figure 7:**
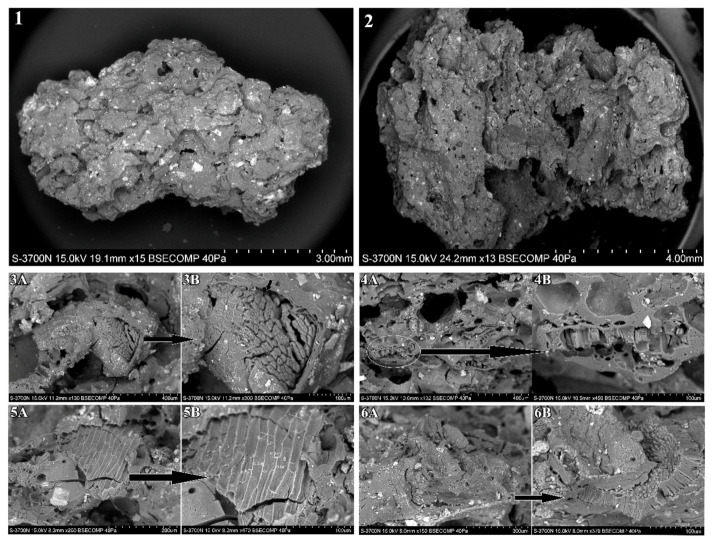
Several SEM micrographs of food fragments from SB: (**1**) porridge-like fragment from s.u. 130 made with coarse plant flour; (**2**) bread-like fragment from s.u. 125 made with fine plant flour; (**3A**) SEM micrograph showing fragment of cereal grain embedded in the food matrix; (**3B**) detail of cereal fragment with visible cross-section of epidermis layers (bran and aleurone layers); (**4A**) SEM micrograph showing fragments of aleurone layers embedded in the food matrix; (**4B**) detail of aleurone layer fragment showing the typical rectangular and elongated shape and arrangement of rye/oats aleurone cells; (**5A**) SEM micrograph showing a fragment of cereal bran embedded in the food matrix; (**5B**) detail of bran fragment with visible wheat transverse cells, which differ from those of rye and oats in their elongated shape and prolonged, pointy ends; (**6A**) SEM micrograph showing fragment of legume seed embedded in the food matrix; (**6B**) detail of legume seed with visible hourglass and palisade layer, which measured approximately 30–50 μm in thickness. This is consistent with measurements ranging from 20–70 μm for small pulses such as lentil (*Lens culinaris*) or vetch (*Vicia sativa*).

Microscopic observation under low-powered binocular microscope revealed that five of the six selected fragments were indeed the remains of charred food products, as evidenced by their uniform, porous, and homogeneous microstructure as well as the identification of small, visible plant particles on the surface of the fragments. Further study under SEM allowed for the positive identification of these plant particles as cell tissues, the majority of which were from cereal kernels (grains), pulses, and other non-identified plant species (Figure 7).

In general, the analysed food remains were seen to have a porous and homogeneous internal microstructure. The porosity percentage ranged from 14% to 35%, depending on the fragment; however, the majority of them showed an average of 15% of their surface covered by voids. The voids (air bubbles) were consistent throughout the analysis, with the majority of them being categorised as channel voids, which are normally associated with cooking techniques such as boiling or steaming rather than baking or roasting, which encourage the creation of great amounts of closed voids [53,54].

Two fragments from s.u. 130 and one sample from s.u. 107 presented porous and semi-porous microstructures, respectively, which were formed by a relatively large amount of channel voids (holes). In combination with this, the particles seen in these three fragments were relatively large fragments of cereal grains and pulses (>500 μm on average), suggesting the use of coarse flour for their preparation. In contrast, the fragments from s.u. 104B and 125 differed from the rest in the abundant presence of closed voids ranging in size between 100 μm and 250 μm, which is typical of baked foodstuff such as bread-like products. In addition, the particles seen in these two fragments were of smaller size (<500 μm on average), which suggests the use of finer flour.

Due to the disparity in preservation among the analysed food fragments, the identification to the genus level of the visible particles was only possible in a limited number of samples. The quantity of particles identified within the food microstructures also varied from fragment to fragment, most likely due to the differences in preparation and cooking techniques used. The average number of particles identified per 50× magnification SEM capture from each food fragment was 2.8, which is relatively consistent with the average number of particles identified for experimental and archaeological bread-like products [54]. This said, there were marked differences in the number of particles identified in the samples from s.u. 104B and 125, with only 1 or 2 particles identified, and the samples from s.u. 107 and 130, in which between 3 and 6 particles were identified on average.

The totality of particles identified in the food fragment microstructures were of plant origin and mainly derived from cereal grains and occasionally from pulses. Amongst the identified plant particles, the fragments of cereal grains and the patches of epidermal layers from the cereal kernel (bran and aleurone layers) were the most abundant.

The food fragments from s.u. 107 and 130 contained the most plant particles, with a variety of cereal and pulse tissues identified within their food matrix. Fragments of cereal grains with visible epidermis and endosperm cell layers were the most predominantly identified during the microscopic analysis of the recovered food remains (Figure 7). These ranged in size between 300 and 1000 μm and had visible layers of bran epidermis (transverse and longitudinal cells) as well as aleurone cell layers and endosperm cell tissues. Slight differences in the shape, arrangement, and size of the transverse, longitudinal, and aleurone cells suggest the presence of a variety of cereal types, especially for the two fragments analysed from s.u. 130, where at least three cereal types were identified: barley, wheat, and potentially rye/oats. This was particularly evident from the differences in the shape and arrangement of the aleurone cells in combination with the shape and length of the transverse and longitudinal bran cells. While barley species have distinct multiple-celled aleurone layers, the aleurone layers of wheat, rye, and oat species are formed by a single row of cells. Furthermore, wheat species tend to have rounder and smaller cells, while those of rye/oats tend to be more rectangular and elongated in shape [56] (Figure 7). Additionally, while the measurements are very similar, the transverse cells from wheat species differ from those of rye and oats in their elongated shape and prolonged, pointy ends [56,57,58] (Figure 7).

Fragments of pulses were also identified in a food fragment from s.u. 130. These were fragmented as a result of pounding or grinding and mixed in with cereals prior to cooking. The breakage angle of the seeds allowed for the positive identification of the different cell layers in the legume seed coat or *testa*, with especially well-preserved palisade and hourglass cell layers, which measured to approximately 40–50 μm in thickness. Following the identification criteria established by Butler [59] and Winton and Winton [56], fragments of seed coat with palisade layers and hourglass cells whose thickness measures range from 20–70 μm are likely to be derived from small pulses such as lentil (*Lens culinaris*) or vetch (*Vicia sativa*) (Figure 7).

## 5. Discussion

### 5.1. Storage

With or without archaeobotanical data, several studies have identified different types of storage facilities in early medieval sites in Iberia (e.g., [60,61,62,63,64,65,66]). Storage pits were common both in the Christian areas in the north or in the south, under Islamic rule, and the use of *horrea* is also mentioned in written sources from northern Iberia [62].

No such facilities were recorded at SB. Here, storage took place in a building which was destroyed by a fire at the end of the 10th century or in the early 11th century. It was likely constructed mostly on perishable materials which makes it particularly difficult to interpret. Although no structural evidence has been found, the assumption that this building was used for storage is supported by the great amount of grain found in the level where most of the fire remnants were recorded. In the contexts from Phase 2, fruits/seeds continued to be abundant, particularly in the filling of defensive wall 112, and the assemblages were similar to the previous phase, suggesting that debris from the collapse of Phase 1 building, including abundant plant elements, were incorporated in the new construction. As such, the remains from both phases can be interpreted jointly, assuming that most, if not all, were in fact from the fire at the end of Phase 1. The exceptions are the s.u. from the fifth and sixth moments of occupation, which revealed poorer assemblages.

To better understand the storage strategies at the site, it is necessary to address what was stored and where and how was it kept.

In both phases, the assemblages are largely dominated by cereals, mostly rye and oat, and their spatial distribution suggests they were kept in two main areas (Figure 4 and Figure 6). As mentioned before, a strong concentration of fruits/seeds was found in a reduced number of squares. A total of 80.8% of all the fruits/seeds identified were concentrated within 8 m^2^, with particular emphasis in B7-C7 (Tables S3–S5 and Figure 6A,B). Other concentrations were observed but with less remains. These data suggest that cereals were kept in the eastern area of Sector 1, where some space organization seems to have existed as differences in crops distribution have been recorded. Grains of broomcorn millet were identified more to the east, oat and rye grains in the centre, and hulled barley grains slightly further west (Figure 4 and Figure 6C,D).

Five meters away, namely in square E1, a large concentration of fruits/seeds (n = 5127) was identified in s.u. 107, the main fire level, suggesting the presence of another storage space. This hypothesis is strengthened by the occurrence of the only flax seeds in the site, found in nearby squares (F1’and F0). Although adjacent squares D1’/E1’ from s.u. 104 also revealed a considerable concentration of fruits and seeds (n = 6661), this deposit is associated with the last moment of occupation and is likely the result of the remobilization of the sediments.

The fruits/seeds assemblage demonstrates that not all the cereals were stored fully dehusked and ready for consumption. Furthermore, the analyses demonstrate that processed food was kept in the same area as the grains.

Whether the crops were stored partially or fully processed can sometimes be deduced from the amount of weeds and chaff found, but weeds are scarce at SB and provide little information. Corncockle is the most common and is usually a weed from winter cereals. Still, considering that distinct crops are found in the same samples, it is not possible to determine which weeds correspond to which crops. In contrast, the chaff was recorded in significant amounts. However, any analyses on the proportion between the grains and the chaff must take into consideration that inflorescences are less resistant to fire than grains and tend to be underrepresented (e.g., [67,68,69]), the degree to which depends on several factors, such as temperature and time of exposure, which are difficult to assess. Nonetheless, the differential presence of chaff gives us the indication that distinct species were kept in different processing stages (Tables S3–S5).

Chaff from free-threshing cereals, such as rye and naked wheat, was scarce considering the amount of grains that were found. It is not surprising as grains are easily released through threshing, which is frequently carried out outside the settlement (e.g., [10,67,70,71,72]). Even considering the differential preservation of chaff and grains, it is likely that both cereals were stored fully processed.

In contrast, after threshing, oat, barley, and millet need further processing in order to free the grains [67,70], and at SB many grains of these species were found partially husked. Regarding barley and broomcorn millet, fully hulled grains and grains with the remains of husks still attached are dominant over the clean grains: 5676 vs. 4019 in the case of barley, and 6339 vs. 5898 for millet. These data suggest that both crops were stored hulled.

The case of oat is more complex. The amount of fully hulled grains and grains with some husk remains attached (n = 9751) comprises 19.2% of the grains found. Plus, 615 floret bases without grains were found. The percentage of hulled grains is far lower than those of barley and broomcorn millet, raising doubts about whether the oat grains were preferably stored hulled or if hulled and fully dehusked grains were kept in the same area.

At the settlement of Chadalais (Maisonnais-sur-Tardoire, Haute-Vienne, France), abundant oat grains were found inside an 11th–12th century pit, and although less than 10% were found with husks, the assemblage was still interpreted, even if with reservations, as evidence of the storage of hulled grains [73]. This may have been the case at SB. Any possible preservation bias of barley, broomcorn millet, and oat chaff should be addressed with experimental work in the future.

Such partial processing of cereals is the most effective way to preserve the grain in storage [10], but it is also possible that the barley, broomcorn millet, and oat were being kept hulled to be sown later. The rarity of foxtail millet grains does not allow proper considerations regarding this crop.

The possibility that these cereals were going to be used as fodder must be considered as it would render dehusking unnecessary. The use of barley, oat, and millet as fodder is mentioned in historical documentation from different periods as well as in ethnographic studies [7,74,75,76]. Some archaeobotanical work, such as the one conducted at the medieval site of Zornoztegui in northern Spain, expresses this hypothesis, although not without doubts, regarding barley and oat [77]. However, these crops were likely consumed by humans on a regular basis and the food remains from SB support this assumption. Although the use of oat in these food preparations is not conclusive, and millet has not been detected yet, barley, wheat, pulses, and a third cereal (oat or rye) have been detected.

As such, barley and broomcorn millet, and probably also oat, were very likely stored hulled. If they were going to be used for human consumption, their processing would have occurred on a daily basis. In contrast, naked wheat and rye seem to have been stored as clean grain. These different strategies may be connected with morphological differences in these species, which demand distinct processing procedures in order to free the grain. Some can demand a great deal of time and effort, or even labour, which could have been also a determinant factor when deciding how to store cereals [78]. Such decisions could also be determined by the use given to such crops (e.g., for sowing or human or animal consumption), although the ethnographic data suggest that is not always the case [79]. In Sector 1, evidence was found of domestic animals, namely an ox molar (*Bos Taurus*) and an iron spur (suggesting horse presence) [26].

The presence of made/cooked food products demonstrates how versatile storage areas can be. The distinct combination of two types of particles (coarse and fine) with two kinds of air bubbles or voids which are linked to two very different cooking processes (channel voids for boiling vs. closed voids for baking) suggest that the analysed food fragments belong to two categories of cereal products: a porridge-like product or gruel and a bread-like product. This was clearly exemplified in the samples from s.u. 125 and 130 (see above), with the former being the remains of a bread-like product and the latter that of some type of porridge or gruel or even a soup. It is likely that the remains of bread are those of some type of flat bread rather than leavened or fermented bread, as evidenced by the relatively low porosity percentage (between 10 and 20%) of the microstructure. Porosity percentages higher than 30% have been associated with leavening or the addition of yeast, which promotes the creation and multiplication of gas pockets or air bubbles in the dough during the fermentation and baking process [54,80].

Food remains were recovered from three contiguous squares (B7-C6-C7), including levels from the fire and sediments remobilized and reused in the wall, where we find abundant fruits/seeds. Thus, the remains of two different types of ready-to-use foods are found together with fully processed (rye and wheat) and partially processed (oat, barley, and broomcorn millet) grains, as well as a few pulses and fruits. These distinct elements must have been kept separately in some way, either horizontally, in the house floor, vertically, using shelves or hanging bags in the ceiling, or in a combination of both solutions. This is also suggested also by some differences in the distribution of the taxa (Figure 4 and Figure 6). Although there is no direct evidence of containers, barriers, or furniture, these were likely made of perishable materials. We cannot exclude the possibility that some of the charcoal identified came from such elements, as in the 11th-century granary of La Gravette (L’Isle-Jourdain, Gers, France) [81]. However, at SB, that was not perceived in the spatial distribution of charcoal, and most of the remains came from trunks or other large pieces of oak and chestnut trees, likely used in the building itself.

Still, the grain was not likely to have been stored loose, and the use of textile or wood containers seems reasonable. This type of storage has been recurrently mentioned in several chronological/cultural contexts (e.g., [82,83,84]). At the medieval site of Tremona Castello (Switzerland), braided plant fibres were identified in a storage context, suggesting perishable containers were used to keep crops and protect them against the high humidity of the region [85]. Although no such evidence has been found at SB, a bronze hoop was found in association with the fruits/seeds. It is possible that it was used to hang some container from the ceiling [26].

### 5.2. Crop Diversity

SB provided an outstanding archaeobotanical assemblage, including fruits/seeds, wood, and food remains. However, the cereals clearly stand out (Figure 5). Oat and rye are predominant, while hulled barley, broomcorn millet, naked wheat, and foxtail millet are less common. Although it is impossible to distinguish wild and cultivated oat based solely on grain morphology, the presence of floret bases testifies for the presence of cultivated taxa, *Avena sativa*, or *Avena strigosa*. Distinguishing the floret bases of these two taxa is sometimes possible; however, the morphological characteristics of both species frequently overlap, which is aggravated by differences between the first and second florets [35,40,41,42]. Some floret bases from SB revealed characteristics consistent with *A. strigosa*, such as small and pointed ends, as well as some distance between the lemma base and the scar. Still, these were recorded only in a few better preserved remains, and even then, some variability was observed, and doubts remain, because of which a cautious designations of *A. sativa/strigosa* was adopted. The dominance of oat alongside rye suggests it was not a weed (e.g., [38,86]). SB thus represents a good example of the important dietary role cereals and cereal preparations such as bread and porridge played in the medieval period in the Iberian Peninsula, and specifically during the 10th and 11th centuries AD. Ethnoarchaeological and historical sources corroborate this with mentions of porridge-like products, which included a variety of gruels and soups, as the main cereal product consumed during this period [87,88,89]. Bread, and in particular flat breads, despite being commonly consumed during this time, especially amongst the Al-Andalus populations [90], did not seem to be the primary cereal product until approximately the 15th century AD [88,89].

Considering that most of the remains from SB were originated by a single destructive event, it is impossible to determine whether this assemblage is representative of the site occupation in the longue durée; nonetheless, this study reveals that the people who lived at SB relied on a diversity of cereals, together with some pulses and fruits, which they used to obtain different food products. Coincidentally, the pottery assemblage shows the record of pans for boiling food or cooking porridge and soups, as well as a dish for cooking flatbread.

SB is located amidst granite tors in a rugged mountainous terrain with only a few even areas with abundant soil. Unsurprisingly, the most common cereals recovered are undemanding crops that can thrive in thin and acidic soils, namely oat and rye. So, whether representative or not, their abundant presence makes sense when taking into account the location of the site. Barley also fits well in this strategy. Wheat and, eventually, millet and flax were more easily cultivated near the stream located only around 1.5 km away, where soils and water were more abundant. Pulses and flax were likely cultivated in small garden plots where lack of soil and water could be compensated with human labour, only possible in short cultivation areas, considering that the community inhabiting SB was likely small.

The role of fruits is difficult to assess. They are sporadic at the site, but there is possibly a preservation bias. These are frequently underrepresented in the archaeobotanical record, being more frequent in waterlogged contexts [15]. Besides, they could have been kept in different area within the site. Grapevine, sweet cherries, and chestnuts are native to western Iberia. Despite it being impossible to determine whether the remains found at SB came from wild or cultivated plants, as the few remains are insufficient to conduct morphometric studies, they were likely cultivated given the chronology of the site.

The diversity of crops recorded in a context of environmental constraints to agricultural activities may be related to a strategy to build resilience [91]. Despite evidence of extra-regional contacts, considering the general scenario of instability and insecurity, these communities were likely largely self-sufficient. Having several winter and spring crops suitable for distinct plots would be crucial to take full advantage of the surrounding land, particularly when suitable fields are scarce, to prevent food shortage.

All the crops identified are common in medieval sites in Iberia, although their relative proportions vary within regions and sites [15]. They are also found in western Iberia, usually in small amounts, probably due to the small number of systematic studies. These include wild or cultivated oats as well as rye, which are recorded in several medieval sites, usually in very small numbers [15,17,20,24]. Still, the increasing relevance of rye in medieval times argued for many in European regions [81,92] surely also occurred in the northern and central mountain areas of Portugal, according to the few archaeobotanical and written data [7,8,91,93].

Most of the crops have already been recovered in the Mondego region, although only at SB is there a clear predominance of rye and oat. At São Gens (Celorico da Beira), broomcorn millet is dominant, followed by rye. Barley and naked wheat are rare; a single grain of wild or domestic oat has been recorded and no domestic pulses or fruits were found. However, here too, the data come from a single building that collapsed after a fire, being its representativeness unknown [14,94]. A single endocarp of sweet cherry has been identified in another sector of the site [23]. At Penedo dos Mouros (Gouveia), a large concentration of faba bean has been recorded, as well as some grains of free-threshing wheat [21]. Again, these remains come from a sole context connected with a destructive event. Thus, although remains are fairly abundant in the three sites, none of which is as abundant as SB, the data from each site are highly determined by specific contexts and are spatially and chronologically restricted. Moreover, the sampling effort was far higher at SB. As such, the absence or rarity of oat in the other sites, contrasting with its abundance at SB must not be overrated, as is the case with the exclusive presence of foxtail millet, lentils, peas, and grass/red peas, as well as grapevine and chestnut. The two fruits have been registered in other chronologies in the region. Pips were recorded at Vila do Touro (Sabugal) [95] and Torre dos Namorados (Fundão) (unpublished) dating to the Iron Age and the Roman period, respectively. As for chestnut, pollen analyses document its presence in the Mondego region at least since the Roman period [96]. At SB, the wood identified in the charcoal analysis testifies for the presence of chestnut and cherry trees in the surroundings. Even though vine was not identified in the charcoal analysis it cannot be ruled out that vines were locally cultivated. The absence of wood in sites with pips is common in several chronologies, even where facilities and vessels related to wine production and storage were identified (e.g., [97]).

Written sources in central Portugal mention that taxes were mostly paid with wheat, without specifying which species [7]. Oat and rye were probably less valued, but at SB, they seem to have been particularly relevant. Still, the written sources are a few centuries more recent, and political and social reality in the region at the 10th–11th century was very different. Moreover, we do not know the role of SB in the local settlement.

## 6. Conclusions

Archaeological investigation is the best way to obtain information regarding small rural communities away from the main centers of power, otherwise they are almost undetectable in the historical records. In this context, the development of archaeobotanical analysis is fundamental to address matters such as agriculture choices or storage strategies and thus obtain information which is crucial to understanding food production and consumption beyond the restricted information recorded in the written documentation.

The results from SB contributed to closing a gap regarding early medieval agriculture and consumption of plant-based products in western Iberia. Most of the available written sources are displaced in time and refer to a specific relationship between central authority and their vassals, providing little information suitable to the understanding of small settlements such as SB, particularly in the context of the instability originated by the frequent conflicts between Muslims and Christians. Even though the archaeological data suggest that the people from SB and the other sites in the region contacted with both sides, it is difficult to understand their involvement in those dynamics. In fact, the panorama observed in central Portugal points to some level of local engagement, where past communities could have shaped their surroundings and freely chosen the crops to sow [2,6].

SB provided an assemblage without parallels in the region, due to its dimension and diversity. The amount of cereals found and their distribution suggest the area that was studied was used for storage until it was destroyed by a fire somewhere between the 10th and the 11th centuries AD. The type of storage facilities is unknown but likely made use of perishable containers.

Stored goods were diverse and included cereals, pulses and food products. Grains of oat and rye predominate but other cereals were recurrent, namely hulled barley, broomcorn millet, naked wheat, and some foxtail millet. Fruits and pulses were rarer. Cereals were probably kept in different processing stages: rye and naked wheat would have been stored as clean grain whereas hulled barley, broomcorn millet, and perhaps oat, were likely stored hulled. The identified food products from SB are the first evidence of this type of material for medieval archaeology in western Iberia.

Considering that the assemblage, though large, was originated in a single destructive event, in a particular storage area, it is not possible to determine how representative it is of the agriculture and food choices of the community who inhabited SB. Nonetheless, the study that was conducted showed they relied on a diversity of crops, mostly cereals, that were used to obtain different food products. The priority given to oat and rye, instead of more demanding cereals such as naked wheat, is likely an adaptation to the environmental constraints of the region, namely the rough conditions and lack of deep and fertile soils in the surroundings. Moreover, the diversity of crops identified would assure that this community had the possibility to fully take advantage of its territory in a productive way, in order to prevent food shortage and to guarantee its self-sufficiency in uncertain times.

## Figures and Tables

**Table 1:** Radiocarbon dates obtained from Sector 1 at SB. Calibration Oxcal 4.4.2 software [27], Intcal 20 calibration curve [28].

S.U.	Description	Context	Material	Lab. Reference	14C Age (yr B.P.)	Calibrated Age A.D. (2 σ)
**107**	Destruction layer	Habitation structure 09	*Vicia faba* (seed)	Wk-40079	1040 ± 21	987–1032 (95.4%)
**125**	Filling layer	Debris within Wall 112	*Secale cereale* (grain)	Beta-46513	1170 ± 30	772–901 (73.9%) 916–974 (21.6%)
**132**	Clay layer	Hearth 2	*Erica arborea* (charcoal)	Beta-46512	1070 ± 30	893–929 (23.6%) 944–1026 (71.8%)

**Table 2:** Overview of the sampling and recovery strategies applied by phase and moment (detailed data in Table S1).

	Collected	Studied									
**Phase 1**	278	198	**Moment**	**Collected**			**Studied**			**No. of S.U.**	**No. of Squares**
			**1st**	15	Handpicked	15	15	Handpicked	15	2	8
			**2nd**	263	Handpicked	155	183	Handpicked	155	6	49
					Sediment	108		Sediment	28		
**Phase 2**	268	241	**3rd**	7	Handpicked	1	7	Handpicked	1	3	4
					Sediment	6		Sediment	6		
			**4th**	125	Handpicked	81	99	Handpicked	81	8	24
					Sediment	44		Sediment	18		
			**5th**	8	Handpicked	4	7	Handpicked	4	3	5
					Sediment	4		Sediment	3		
			**6th**	128	Handpicked	120	128	Handpicked	120	8	64
					Sediment	8		Sediment	8		

**Table 3:** Fruits/Seeds of crops and likely cultivated species.

**Occupation**	Phase 1							Phase 2																
**Moment**	1st		2nd					3rd			4th					5th			6th					
**Structure**	HS9		HS9					W112			W112					H1	H2	GM	W112			SS123	PHS	
**S.U.**	115	116	106	107	108	109	137	136	139	140	124	125	130	131	134	126	132	133	104	104A	104B	118	102	121
**Cereal (grain)**																								
*Avena* sp.	42	1	9	36,609	16		130	946	833	215	165	5166	3948	309	1	20	1	1	1050	1076	143		143	1
*Hordeum vulgare*	22	2	48	7248	9		5	13	99	17	43	666	506	40		9	5		324	221	284		134	
*Panicum miliaceum*			8	8345			44	299	210	60	71	978	487	74		11	7	2	1490	128			3	20
*Setaria italica*				123					6		4	19		6										
*Setaria* sp.				9																				
Panicoideae				1128				17	30		29	299	171	29		2	1		179	42	2			5
*Secale cereale*	84	6	204	34,204	25		61	776	649	136	130	4471	3679	436	1	10	4	1	1989	439	565	1	362	4
*Triticum aestivum/durum*	12	1	27	965	6		4	22	25	6	9	498	38	107		2	1		67	21	26	1	14	3
*Triticum* sp.				4								5												
Triticeae		2	3	10,424			18	234	210	44	233	1338	1346	132		8	2		745	416	58	1	91	1
**Cereal (chaff)**																								
*Avena sativa/strigosa* (floret base)				842			11	10	40		4	127	173	25						54	10		2	
*Hordeum vulgare* (lemma base)				689				31	3			96		2						19	17		4	
*Hordeum vulgare* (rachis segment with 1 node)				48																4				
*Panicum miliaceum* (lemma/palea)				32					8															
*Setaria italica* (lemma/palea)									3					2										
*Secale cereale* (rachis segment with 1 node)				741				131	48	13	19	213	406	5		2		1	16	76	1			1
*Secale cereale* (rachis segment with 2 nodes)				18				19			2													
*Triticum aestivum/durum* (rachis node)				122																4				
**Fabaceae**																								
*Lathyrus cicera/sativus* (seed)				71	1					2		14		1										
*Lens culinaris* (seed)				18																1				
*Pisum sativum* (seed)	1		1	412	1							8		2						1	3		2	
*Vicia faba* (seed)	10		1	238							1	18		1						1	7		9	
*Vicia/Lathyrus* (seed)			1	250				8	9			39		4			2		12	4	2		1	
*Vicia/Lathyrus/Pisum* (seed)			1	272	1	1					1	58	4	8		1				4	1		3	
**Fruits**																								
*Castanea sativa* (fruit)				2																				
*Prunus avium/cerasus* (endocarp)				2																			1	
*Vitis vinifera* (seed)	1			101				24					8						4		1			
*Vitis vinifera* (pedicel)				7				7	3				17							1				
*Vitis vinifera* (drupe with seed)															2									
**Oil/Fiber plants**																								
*Linum* sp. (seeds agglomerated)				6																				

**Table 4:** Charcoal results from SB.

**Phase**	1										2																							
**Moment**	1st		2nd						**Total**		3rd			4th								5th			6th								**Total**	
**S.U.**	115	116	106	107	108	109	128	137	N	%	136	139	140	124	125	125A	129	130	131	134	138	126	132	133	102	103	104	118	120	121	104A	104B	N	%
*Acer* sp.				2					2	0.02								1															1	0.01
*Alnus* sp.				67					67	0.68				1	36			10							1					2	2		52	0.59
*Arbutus unedo*		4	56	460	58			15	593	6.03	55	18	6		105			11	2	13	2	16	13		32	20	27	2	2	32	23	12	391	4.46
*Castanea sativa*	15	60	25	2893	195	119	4	55	3366	34.22	228	109	33	62	1173	4		154	163	133	1	19	13		112	15	60	78	40	163	445	134	3139	35.81
*Castanea/Quercus*	4	18	27	1017	51	6			1123	11.42				29	194	2		14	13			20	22		103	13	11	9	5	66	73	20	594	6.78
*Cistus* sp.			1	22				8	31	0.32	17	27	17		4			5		2					3		31				1		107	1.22
*Erica australis/arborea*	5	21	27	409	105	10			577	5.87	19	1		7	98	4	1	13		6		26	20		75	8	15	30	3	51	46	9	432	4.93
*Erica scoparia/umbellata*			2	25					27	0.27	2				6											2	5	1			1		17	0.19
*Erica* sp.		5	19	329	46	3		12	414	4.21	17	10		3	49			20	2			23	19	5	43	4	5	3		28	33	6	270	3.08
Fabaceae		3	2	126	20			1	152	1.55	9		1	5	56			17	3	2			5	2	52	3	18	2	1	4	11	2	193	2.20
*Frangula alnus*				2					2	0.02																								
*Fraxinus* sp.		1		14	4				19	0.19	1	1	1		12			2						1			2			4	2		26	0.30
*Olea europaea*															1																		1	0.01
*Pistacia* sp.				3					3	0.03																								
*Pinus* sp.												1																					1	0.01
*Prunus avium/cerasus*				44					44	0.45	3	22			8			2	5	3	9									20	5		77	0.88
*Prunus* sp.	12	53	1	163	120	15	1		365	3.71	3	16		23	64				1			2	1		70		7	42	8	60	42	18	357	4.07
*Quercus* sp.	1	3	11	148	2				165	1.68				4	24			1	1			3	6		8	5		1		16	6		75	0.86
*Quercus* sp. deciduous	8	29	192	1145	133	26	1	48	1582	16.08	167	93	51	15	297	53	2	56	1	36	2	35	7	6	286	55	111	135	44	308	208	20	1988	22.68
*Quercus* sp. evergreen		3	10	100	15	1		3	132	1.34		3		2	8			2					1		7	2	2	2		10	4	7	50	0.57
*Rhamnus/Phillyrea*			1	3	3				7	0.07	2				2			1		1		1					1						8	0.09
Rosaceae Maloideae				7					7	0.07		2			1																3		6	0.07
*Salix* sp.				2	6				8	0.08								1		1					2								4	0.05
*Salix/Populus*				1					1	0.01																								
*Taxus baccata*				52					52	0.53					1																2	12	15	0.17
Undetermined		2		30					32	0.33				3	4							1			3			2		2			15	0.17
Dicotyledon	1	23	54	852	88	20		8	1046	10.63	27	22	7	26	353			28	21	2	1	28	44	5	86	18	46	18	3	119	82	7	943	10.76
Monocotyledon				1	2				3	0.03					3																		3	0.03
Bark				16					16	0.16																								
**Total**	46	225	428	7933	848	200	6	150	9836	100	550	325	116	180	2499	63	3	338	212	199	15	174	151	19	883	145	341	325	106	885	989	247	8765	100

## Data Availability

Not applicable.

## Supplementary Materials

The following supporting information can be downloaded at https://www.mdpi.com/article/10.3390/plants11040471/s1, Table S1: general inventory, Table S2: subsampling strategy, Table S3: fruits/seeds results per s.u., moment and phase (units only), Table S4: fruits/seeds results per sample, Table S5: fruits/seeds results per square and s.u., Table S6: fruits/seeds per litre (units and only samples from 2016 campaign), Table S7: charcoal results (Phase 1), Table S8: charcoal results (Phase 2), Table S9: tree ring curvature, vitrification, and radial cracks per taxon, Table S10: analysed charred food products.
